# Spatial Characterization of Soybean Yield and Quality (Amino Acids, Oil, and Protein) for United States

**DOI:** 10.1038/s41598-018-32895-0

**Published:** 2018-10-02

**Authors:** Y. Assefa, N. Bajjalieh, S. Archontoulis, S. Casteel, D. Davidson, P. Kovács, S. Naeve, Ignacio A. Ciampitti

**Affiliations:** 1 Department of Agronomy, Kansas State University, Manhattan, KS United States; 2 FIRST Seed Tests, 562 S Prairie St., Cary, IL United States; 3 Department of Agronomy, Iowa State University, Ames, IA United States; 4 Department of Agronomy, Purdue University, West Lafayette, IN United States; 5 Illinois Soybean Association, Bloomington, IL United States; 6 Department of Agronomy, Horticulture, and Plant Science, South Dakota State University, Brookings, SD United States; 7 Department of Agronomy and Plant Genetics, University of Minnesota, St. Paul, MN United States

## Abstract

Continued economic relevancy of soybean is a function of seed quality. The objectives of this study were to: (i) assess the spatial association between soybean yield and quality across major US soybean producing regions, (ii) investigate the relationship between protein, oil, and yield with amino acids (AAs) composition, and (iii) study interrelationship among essential AAs in soybean seed. Data from soybean testing programs conducted across 14 US states from 2012 to 2016 period (n = 35,101 data points) were analyzed. Results indicate that for each Mg ha^−1^ yield increase, protein yield increased by 0.35 Mg protein ha^−1^ and oil yield improved by 0.20 Mg oil ha^−1^. Essential AA concentrations exhibit a spatial autocorrelation and there was a negative relationship between concentration of AA, protein, and oil, with latitude. There was a positive interrelationship with different degree of strength among all AAs, and the correlation between Isoleucine and Valine was the strongest (r = 0.93) followed by the correlation among Arginine, Leucine, Lysine, and Threonine (0.71 < r < 0.88). We concluded that the variability in genotype (G) x management (M) x environment (E) across latitudes influencing yield also affected soybean quality; AA, protein, and oil content in a similar manner.

## Introduction

Soybean [*Glycine max* L. (Merr.)] production area in the United States (US) increased from 29.3 to 36.2 million harvested hectares from 2000 to 2017^1^. A primary driving factor for the growing trend in soybean production is its economic importance due to versatile end use, seed protein, and oil^2,3^. Soybean serves as an oil seed crop, feed for animals, protein source for human, and biofuel feedstock^4^. Production of high protein requires less land and exhibits a smaller carbon footprint than producing the same protein from animal or plant sources^5^. Maintaining soybean economic advantage will require study of the seed quality factors and their degree of linkage with genetics, environment, and management (G × E × M). Seed quality composition for a given variety is determined by the G × E interaction^6^.

Seed amino acid (AA), or protein at large, composition is among the main factors determining soybean quality^7^. Soybean is a relatively low-cost protein source for human and animal nutrition and its protein is composed of all essential AAs^8^. Typically, soybean seed contains 40% of protein, far greater than any other vegetal protein sources such as beans (*Phaseolus vulgaris* L.) and peas (*Pisum sativum*)^5^. Together, essential AAs (isoleucine, histidine, leucine, lysine, methionine, phenylalanine, threonine, tryptophan, and valine) and conditionally essential AAs (arginine, cysteine, glutamine, tyrosine, glycine, ornithine, proline, and serine) comprise about 20% of the soybean seed protein^5^. For the same crop, the total sulfur (S) containing AAs (TSAA), cysteine and methionine, compose less than 1.5% of the total protein, below the level to meet the human daily dietary recommendation^9^.

Environment has been indicated as one of the most important factors determining AA composition for US^10^, Argentina^8^ and Brazil^11^. Lowest concentration of AAs was reported for soybean meals from northern to southern regions in US^12^. Likewise, an increasing trend in soybean AA concentration was documented from northern to southern regions in China^13^. From the protein standpoint, Rotundo *et al*.^14^ reported a greater protein concentration in soybeans from southern US regions. Comparing soybean meals from US, Brazil, and China, several researchers^12,15,16^ concluded that environmental conditions greatly impacted seed composition. Among the main environmental factors affecting protein and AAs composition were temperature, solar radiation, water availability, and soil nutrient supply^8,17–24^. Regarding soil nitrogen (N) availability, Krishnan *et al*.^25^ reported that N abundance reduced sulfur AA content in soybeans. Crop management such as irrigation^7,26^ and other factors such as genetics (crop maturity and variety), phenology, node position, and disease also affected AAs and overall seed composition for soybeans^27–29^. Different locations will experienced different environmental conditions which may give rise to a large variability of soybean yield and quality across regions.

**Figure 1:**
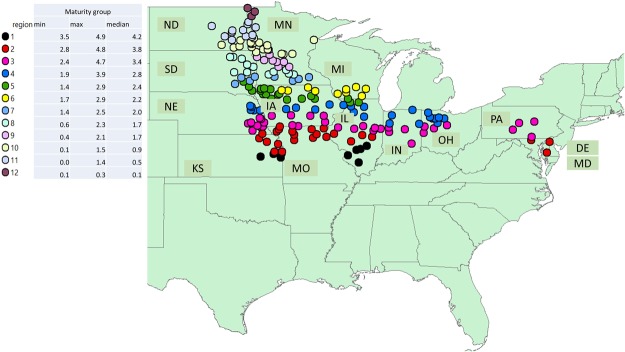
Locations of soybean testing programs within the 14 United States (US) states where experiments were conducted from 2012 to 2016 period. Dots represent city locations and colors represent regions with similar soybean maturity group in test. The minimum, maximum, and median maturity groups for the region are indicated in the legend.

A negative relationship has been documented in the scientific literature between yield and protein or AA concentration^30–32^. However, the concentration measures abundance of a quality trait relative to other competing components. Thus, it is not clear whether the negative relationship between protein and yield is due to a decrease in protein concentration *per-se* or due to an increase in the composition of other seed components when increasing yield. It is indicated that, even though there is a negative relationship between protein concentration and yield, protein yield and seed yield correlate positively^30^. Mourtzinis *et al*.^33^ also reported a positive correlation between seed yield with protein and oil content. Variability in US soybean seed composition has been already characterized by several researchers^3,34–36^ and, more recently, for protein and oil from farmer-provided seed samples by^14^. For AAs, a regional US characterization for the main producing regions is still lacking. Furthermore, the interrelationship among AAs and their individual association with oil and yield is largely unknown. Thus, the objectives of this study were to: (i) assess the spatial association in concentration of AAs for the US soybean producing regions (Fig. 1), (ii) investigate the relationship between oil and yield with protein and AAs composition, and (iii) study interrelationship among essential and conditional essential AAs in soybean.

## Results and Discussion

### Total essential amino acid, protein, oil, and protein + oil concentration

**Figure 2:**
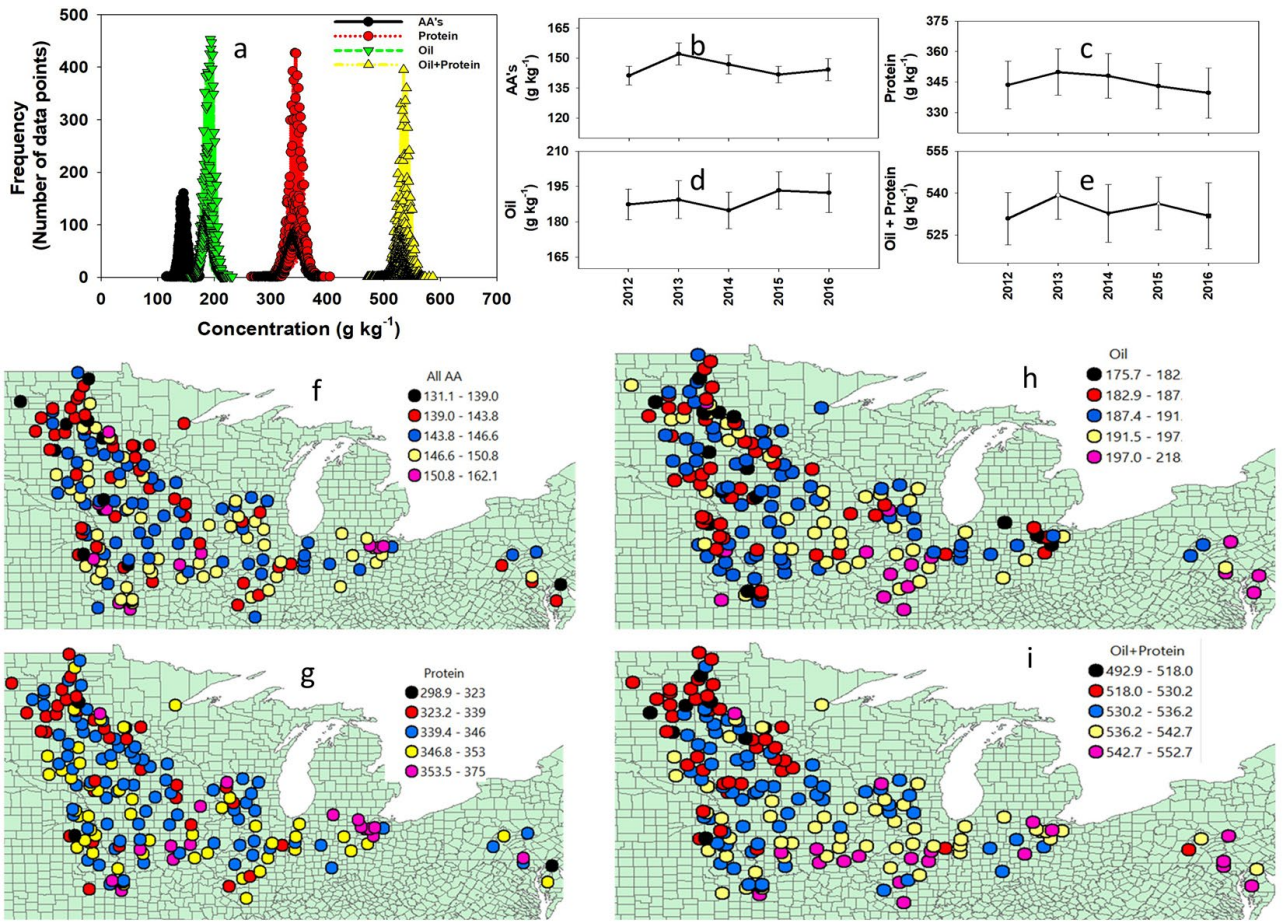
Distribution of the sum of essential amino acid, protein, oil, and oil + protein (**a**), average over each year (**b**–**e**), and trend over 14 major soybean US states (**f**–**i**) during the period 2012 to 2016. Error bars are standard deviation.

The distribution of the sum of 11 essential and conditional essential AAs range from 115 to 174 g kg^−1^, with similar mean and median value of 145 g kg^−1^ (Fig. 2a). The majority of the data (~70%) was concentrated within a 13 g kg^−1^ range, implying a narrow variation for this seed trait. Protein concentration ranged from 266 g kg^−1^ to 405 g kg^−1^, with both mean and median value at 345 g kg^−1^. Similar to the AA, for protein the majority of the data was also concentrated (~70%) within a 24 g kg^−1^ range. Oil concentration ranged from 157 to 232 g kg^−1^, mean and median were 189 g kg^−1^, and the standard deviation was 8.4 g kg^−1^ (Fig. 2a). The sum of protein + oil concentration ranged from 471 to 587 g kg^−1^. Concentration of total AA, protein, and oil varied over time, with AA and protein concentrations peaking in 2013 and with the lowest average oil concentration in 2014 year (Fig. 2b–e).

There was a significant spatial autocorrelation and trend across latitudes for AAs, protein, oil, and oil + protein (Tables 1, 2; Fig. 2f–i). All seed quality traits decreased as latitude increased, however, the negative correlation between latitude and oil + protein concentration was stronger because both protein and oil concentration, individually, decreased across latitude (Table 3).

**Table 1:** Average numbers of sites, varieties per site-years, and range of planting and harvesting dates for soybean trials within each US state between 2012 and 2016 period.

State	Average Number of		Earliest Planting date	Latest Harvesting date
	Sites (year)^−1^	Varieties (site-year)^−1^	…………………….day – month……………………	
Delaware	1	36	26 May	26 Oct
Illinois	16	59	04 May	15 Nov
Indiana	8	51	05 May	29 Oct
Iowa	20	76	12 May	26 Oct
Kansas	8	46	09 May	19 Nov
Maryland	2	36	25 May	14 Nov
Minnesota	12	76	09 May	05 Nov
Missouri	8	32	14 May	11 Nov
Nebraska	8	54	08 May	29 Oct
North Dakota	8	64	09 May	29 Oct
Ohio	8	35	16 May	13 Nov
Pennsylvania	5	53	27 May	15 Nov
South Dakota	12	69	15 May	22 Oct
Wisconsin	4	66	13 May	26 Oct

**Table 2:** Moran’s I Spatial autocorrelation analysis for soybean yield and quality parameters across the major US soybean producing regions.

Amino acid	Observed	Expected	Deviation	P value
Arginine	0.0938	−0.00546	0.0107	0.00
Cysteine	0.0041	−0.00546	0.0105	0.36
Isoleucine	0.0127	−0.00546	0.0107	0.09
Leucine	0.0515	−0.00546	0.0107	<0.001
Lysine	0.0693	−0.00546	0.0107	<0.001
Methionine	0.0418	−0.00546	0.0108	<0.001
Threonine	0.0989	−0.00546	0.0107	0.00
Tryptophan	0.0982	−0.00546	0.0107	0.00
Valine	0.0202	−0.00546	0.0107	0.016
Sum of AA’s	0.0841	−0.00552	0.0108	<0.001
Protein	0.0836	−0.00552	0.0107	0.00
Oil	0.0952	−0.00552	0.0107	0.00
Oil + Protein	0.1966	−0.00552	0.0107	0.00
Yield	0.1232	−0.00561	0.0112	0.00

The null hypothesis of no spatial correlation is rejected for P values less than 0.05.

**Table 3:** Correlation analysis among location (latitude, Lat; longitude, Long), year, amino acid, oil, protein concentration, and seed yield for soybeans.

	Lat	Long	year	Arginine	Cysteine	Isoleucine	Leucine	Lysine	Methionine	Threonine	Tryptophan	Valine	Yield
Arginine	−0.18	0.06	−0.49	1.00									
	<0.0001	<0.0001	<0.0001										
Cysteine	−0.07	0.01	0.24	0.37	1.00								
	<0.0001	0.02	<0.0001	<0.0001									
Isoleucine	−0.08	0.05	0.44	0.37	0.62	1.00							
	<0.0001	<0.0001	<0.0001	<0.0001	<0.0001								
Leucine	−0.10	0.05	−0.16	0.85	0.64	0.56	1.00						
	<0.0001	<0.0001	<0.0001	<0.0001	<0.0001	<0.0001							
Lysine	−0.09	0.05	−0.35	0.82	0.42	0.26	0.82	1.00					
	<0.0001	<0.0001	<0.0001	<0.0001	<0.0001	<0.0001	<0.0001						
Methionine	−0.07	0.06	−0.22	0.66	0.28	0.30	0.63	0.70	1.00				
	<0.0001	<0.0001	<0.0001	<0.0001	<0.0001	<0.0001	<0.0001	<0.0001					
Threonine	−0.11	0.14	−0.36	0.71	0.37	0.14	0.79	0.88	0.65	1.00			
	<0.0001	<0.0001	<0.0001	<0.0001	<0.0001	<0.0001	<0.0001	<0.0001	<0.0001				
Tryptophan	−0.21	0.18	−0.23	0.68	0.35	0.20	0.76	0.75	0.59	0.86	1.00		
	<0.0001	<0.0001	<0.0001	<0.0001	<0.0001	<0.0001	<0.0001	<0.0001	<0.0001	<0.0001			
Valine	−0.05	0.01	0.28	0.44	0.68	0.93	0.62	0.36	0.34	0.24	0.25	1.00	
	<0.0001	0.12	<0.0001	<0.0001	<0.0001	<0.0001	<0.0001	<0.0001	<0.0001	<0.0001	<0.0001		
Yield	−0.20	0.28	0.28	−0.17	−0.04	0.09	−0.08	−0.06	0.05	−0.01	0.03	0.07	1.00
	<0.0001	<0.0001	<0.0001	<0.0001	<0.0001	<0.0001	<0.0001	<0.0001	<0.0001	0.02	<0.0001	<0.0001	
Protein	−0.18	0.11	−0.19	0.84	0.38	0.50	0.75	0.58	0.49	0.50	0.58	0.47	−0.10
	<0.0001	<0.0001	<0.0001	<0.0001	<0.0001	<0.0001	<0.0001	<0.0001	<0.0001	<0.0001	<0.0001	<0.0001	<0.0001
All AA	−0.13	0.07	−0.14	0.87	0.70	0.63	0.96	0.85	0.70	0.78	0.74	0.71	−0.05
	<0.0001	<0.0001	<0.0001	<0.0001	<0.0001	<0.0001	<0.0001	<0.0001	<0.0001	<0.0001	<0.0001	<0.0001	<0.0001
TSAA	−0.09	0.03	0.12	0.55	0.94	0.63	0.77	0.61	0.60	0.55	0.50	0.69	−0.01
	<0.0001	<0.0001	<0.0001	<0.0001	<0.0001	<0.0001	<0.0001	<0.0001	<0.0001	<0.0001	<0.0001	<0.0001	0.03
Oil + Protein	−0.37	0.30	−0.03	0.52	0.33	0.32	0.51	0.41	0.22	0.40	0.48	0.27	−0.02
	<0.0001	<0.0001	<0.0001	<0.0001	<0.0001	<0.0001	<0.0001	<0.0001	<0.0001	<0.0001	<0.0001	<0.0001	0.001
Oil	−0.20	0.22	0.23	−0.56	−0.14	−0.32	−0.45	−0.33	−0.44	−0.23	−0.25	−0.34	0.12
	<0.0001	<0.0001	<0.0001	<0.0001	<0.0001	<0.0001	<0.0001	<0.0001	<0.0001	<0.0001	<0.0001	<0.0001	<0.0001

Pearson correlation coefficients (r) and probability > |r| under null hypothesis (H0): Rho = 0. The correlation analysis had a minimum of 33,526 data points, 4 years (2012–2016) and ~120 locations per year.

### Overall distribution of individual essential AA concentration

**Figure 3:**
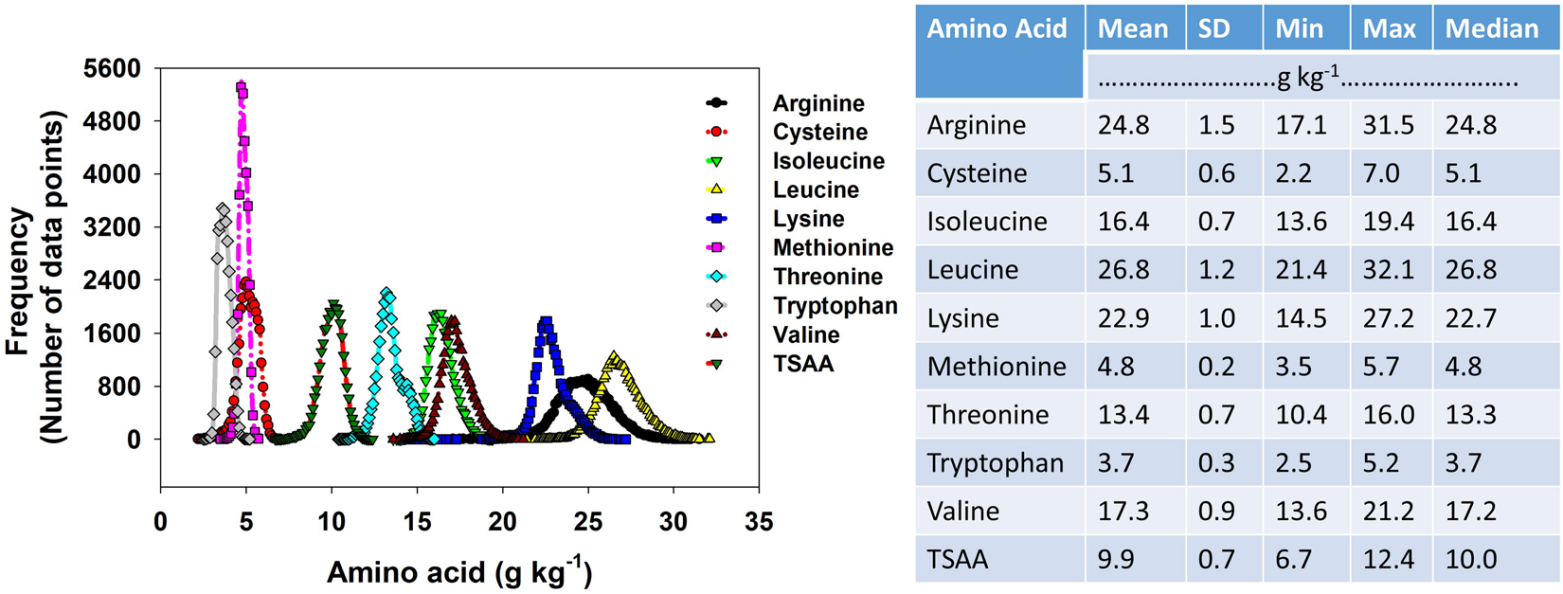
Distribution of amino acid concentrations, expressed in g kg^−1^, in soybeans grown across 14 major soybean producing states during the period 2012 to 2016.

The concentration of each individual essential AA was below 35 g kg^−1^ (3.5% seed weight) with minimum, maximum, and average values varying by AA type (Fig. 3). The overall mean concentration of leucine, arginine, and lysine (26.8, 24.8, and 22.9 g kg^−1^, respectively) was greater than those for valine, isoleucine, and threonine (17.3, 16.4, 13.4 g kg^−1^, respectively). The concentration of the last three AAs was greater than the overall concentration of cysteine, methionine, and tryptophan (5.1, 4.8, 3.7 g kg^−1^, respectively). Median value of each AA did not significantly differ from the mean, with AA distributions close to normal. The narrow ranges between the maximum and minimum values (Fig. 3) or the small standard deviation (<1.5 g kg^−1^) of each essential AA indicate that the impact of any environmental, management, or genetic variation on individual AA concentration of soybeans had only minor (narrow) effect.

The mean concentration of essential AAs reported in this study were slightly lower than the mean AAs values described for the different US regions^10,15,36^. Mean values reported by Karr-Lilienthal *et al*.^12^ for different US soybean collections were greater, in most cases at least 1% superior than the mean values reported in this study. Similarly, our mean values are lower than those reported for Iowa^37^ and Brazil^8^ but greater than those reported by Goldflus *et al*.^11^ for Brazil. Similar to our results, the aforementioned published scientific literature depicted a narrow variation range of AAs among soybean plants depending on performance of crop.

### Environment (Location × Year) impact on essential amino acid concentration

**Figure 4:**
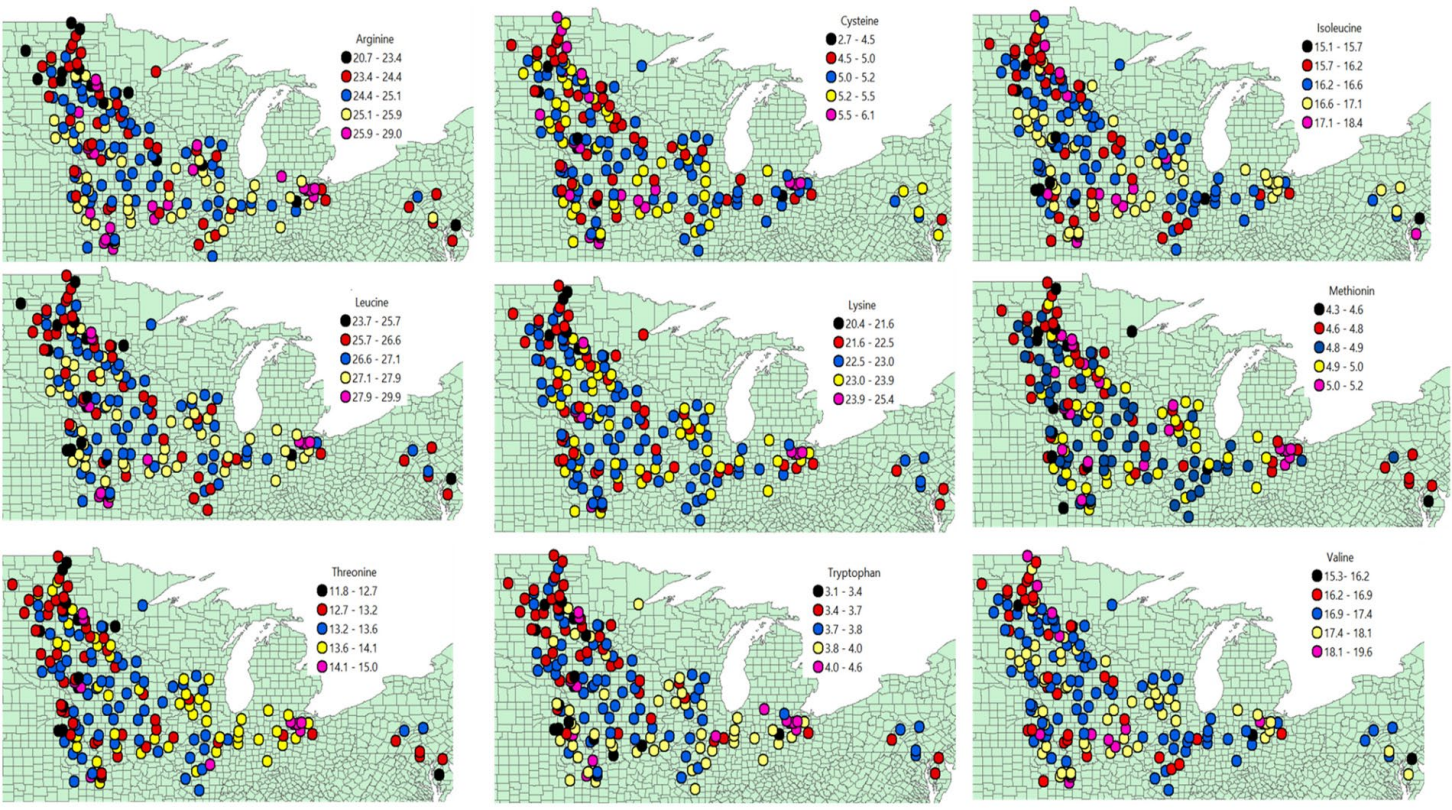
Spatial classification of the average concentration of amino acid in soybeans for the trial across 14 major soybean producing states in the USA from 2012 to 2016 period.

A significant correlation was observed on the concentration of essential AA in soybeans and location (Fig. 4). When a univariate correlation analysis was conducted between AAs with latitude, all essential AAs depicted a negative correlation with a Pearson correlation coefficient ranging from −0.05 for valine to −0.21 for tryptophan. All essential AAs, except valine, showed a positive correlation with longitude with a Pearson correlation coefficient ranging from 0.01 for cysteine to 0.18 for tryptophan. When a spatial autocorrelation analysis was conducted using Moran’s I, a significant spatial autocorrelation was obtained for all except for cysteine and isoleucine (Table 1; Fig. 4). These results suggest a significant impact of geographical location, with relatively lower AA concentration in the North-West than in the South-East US Corn Belt region.

**Figure 5:**
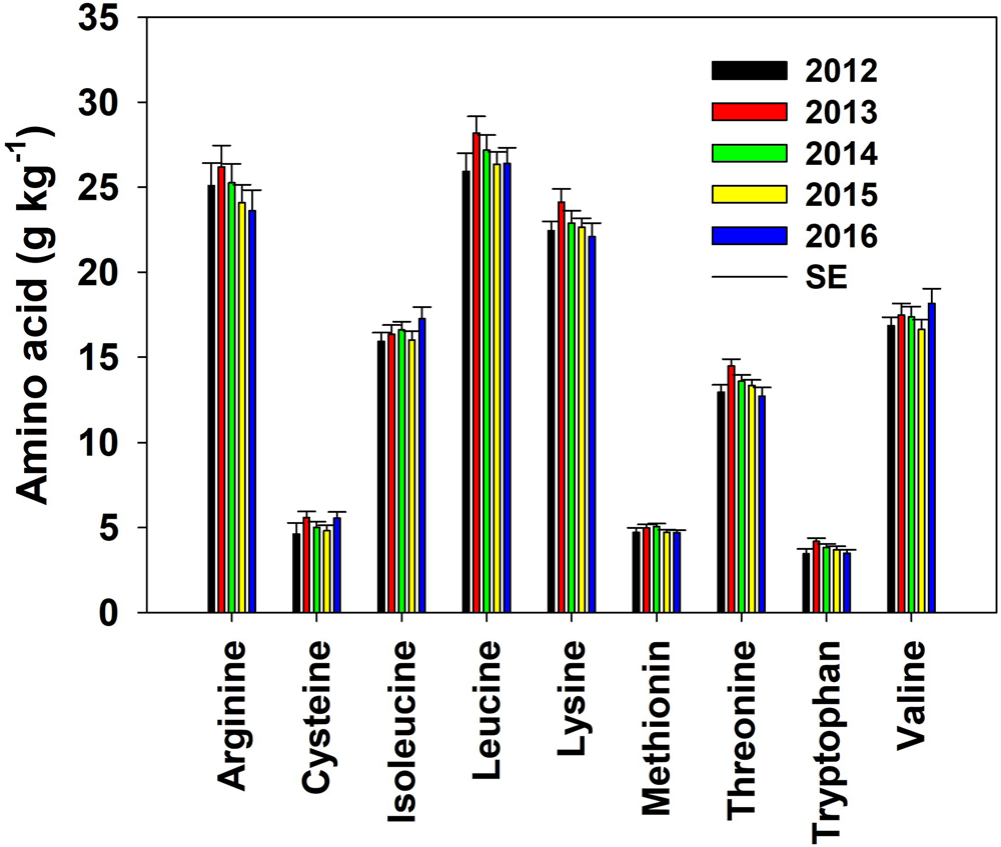
Mean annual amino acid in soybeans for the trials across 14 major soybean producing US states for the years 2012 through 2016.

A significant annual variation was also observed on the concentration of essential AA (Fig. 5). In most cases, the concentration of AA was the lowest in 2012 compared to the other evaluated years, attaining its maximum concentrations in 2013 and 2016. Over the years, concentration of arginine, leucine, lysine, methionine, threonine, and tryptophan tend to decrease but cysteine, isoleucine, and valine tended to increase.

Environment is a major player influencing AA concentration^8^. Variation in location or year result in variations in climatic variables such as temperature, radiation, moisture, and soil nutrients that affect soybean growth and result in different seed composition^11^. In addition, as latitude increases, maturity group gradually changes becoming an additional factor. Attempts were made to study the AA concentration of soybeans across different states within the US and Brazil^8,10–12^ or among countries, US, Brazil, and China^15^ but efforts to look at spatial autocorrelations are scarce in the scientific literature. After a regional comparison on essential and nonessential AA concentration in US soybean meals, Karr-Lilienthal *et al*.^12^ concluded that essential AA concentrations were the lowest for meals sourced from northern regions. The current spatial analysis is in agreement with the literature, but adds that there is variation in the degree of spatial correlation for the AAs considered. The correlation between AAs and latitude is confounded with the effect of the environment (weather × soil and length of the growing season) and with the soybean maturity group between the different US latitudes. Therefore, both environment and genetics play a key role for the spatial trend and autocorrelation reported.

### Maturity impact on essential amino acid in soybean

**Figure 6:**
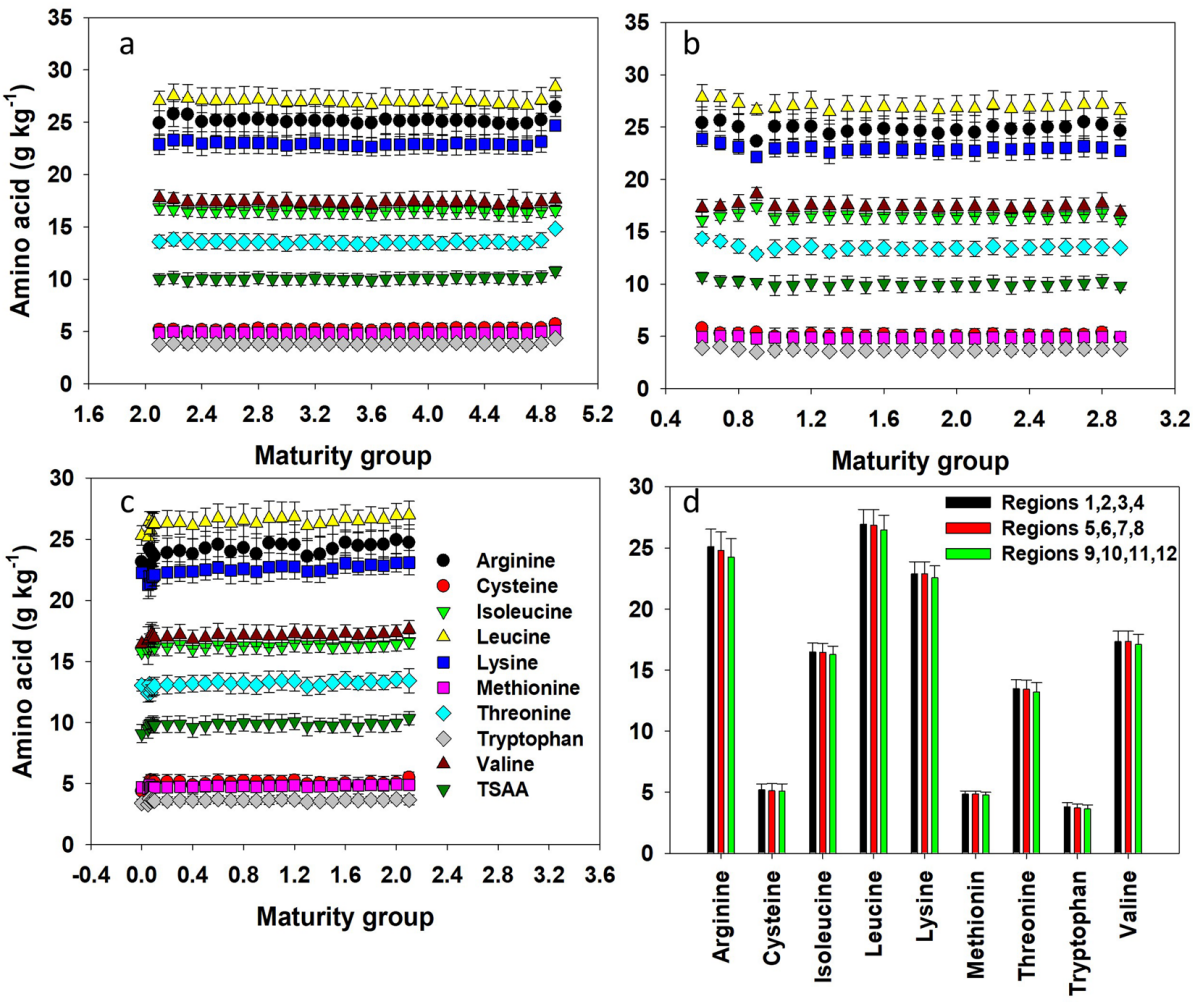
Relationship between percent amino acid in soybeans with maturity group in regions 1, 2, 3, 4 (**a**); 5, 6, 7, 8 (**b**); and 9, 10, 11, and 12 (**c**) and mean amino acid concentration by regions (**d**) for the trial across 14 major soybean producing states in the USA for the years 2012 through 2016.

Since the maturity group factor declines as latitude increases, analysis between maturity group and AA was conducted within a region with similar maturity group. Within regions 1–4, where maturity group ranged from 2.0 to 4.9, a weak negative correlation (Pearson correlation coefficient −0.02 to −0.05) was documented between maturity group and all AAs except tryptophan and cysteine (Fig. 6a). There was no correlation between maturity group and tryptophan, but a weak positive correlation (r = 0.05) between maturity group and cysteine (Fig. 6a). Within regions 5–8, where maturity groups ranged from 0.5 to 3.0, a weak positive correlation (Pearson correlation coefficient 0.04 to 0.11) was obtained between maturity group and all AAs without exception (Fig. 6b). Within regions 9–12, where maturity groups ranged from 0.0 to 2.2, a similar positive but stronger correlation (r = 0.04 to 0.21) than the observed for the regions 5–8 was detected for AA concentration and maturity group (Fig. 6c), except for cysteine. In these regions 9–12, a relatively stronger positive correlation was documented between maturity group and lysine (r = 0.21) and methionine (r = 0.21). Despite detecting correlations between AAs and maturity group within the regional characterization presented above, comparison among regions for AAs revealed a small difference (Fig. 6d). For all AAs, there was no difference across regions but a relative trend of greater AA concentration in regions at lower rather than at higher latitude.

### Relationship among essential amino acid in soybean

**Figure 7:**
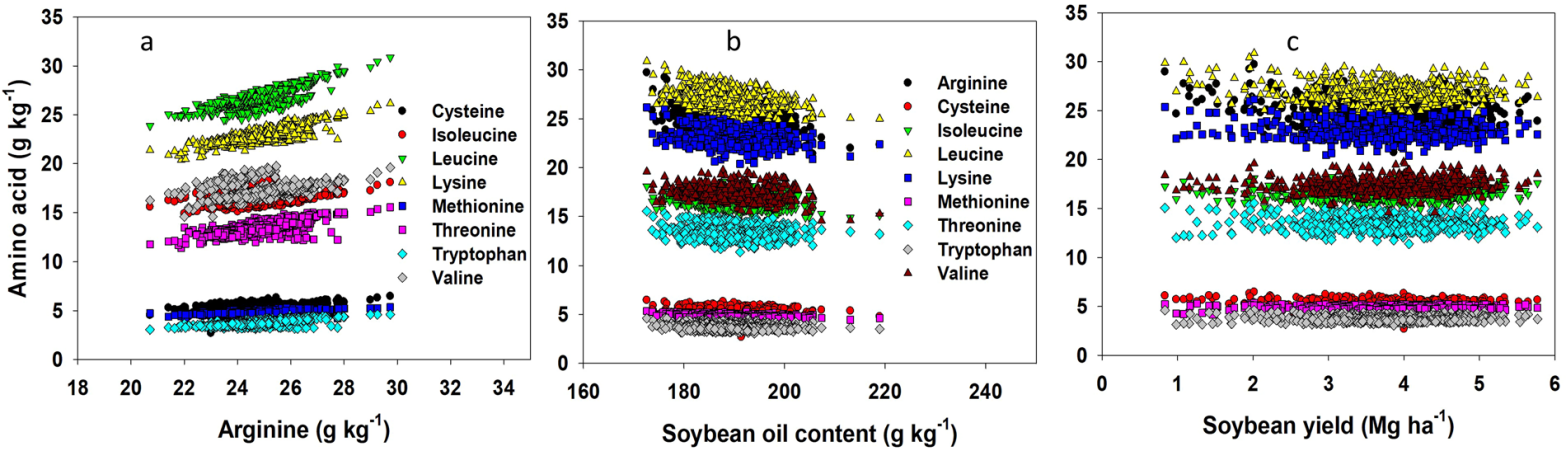
Interrelationship among amino acids in soybeans (**a**) and their relationship with oil (**b**) or soybean yield (**c**) in the trial across 14 major soybean producing states in the USA for the years 2012 through 2016.

A significant positive correlation was evident among all essential AAs (Table 3; Fig. 7a). The strongest correlation was between isoleucine and valine (r = 0.93). The correlation among arginine, leucine, lysine, tryptophan, and threonine was the next strongest (0.71 < r < 0.88); followed by the correlation between arginine, lysine, and methionine (0.66 < r < 0.70); and among cysteine, leucine, and valine (0.62 < r < 0.68). To the extent of our knowledge, not a single study presented in the literature discuss the level of correlation among AAs for soybean crop. The significant and positive correlation among different AAs implies that selection based on one type of AA will come with increase in other AAs, facilitating both breeding and management efforts.

### Relationship between essential amino acid, oil concentration, and soybean yield

There was a significant negative correlation between oil and all essential AAs (Fig. 7b). Arginine, leucine, and methionine presented a relatively strong negative correlation (−0.44 < r < −0.56) with oil concentration relative to the other essential AAs. Similarly, isoleucine, lysine, and valine (−0.32 > r > −0.34), and cysteine, threonine, and tryptophan (−0.14 > r > −0.25) significantly decreased with increasing oil concentration. Similar negative correlation between AAs and oil was recently reported by Mourtzinis *et al*.^13^.

For seed yield, there was a weak and mixed relationship between this trait and AA concentration (Table 2, Fig. 7c). Amino acids such as arginine, cysteine, leucine, lysine, and threonine weakly (−0.01 > r > −0.17) and negatively correlated with yield. Amino acids such as isoleucine, methionine, tryptophan, and valine weakly (−0.01 > r > −0.17) but positively correlated with yield. Mourtzinis *et al*.^13^ also found a weak positive correlation (r = 0.11) between essential AA concentration and yield.

**Figure 8:**
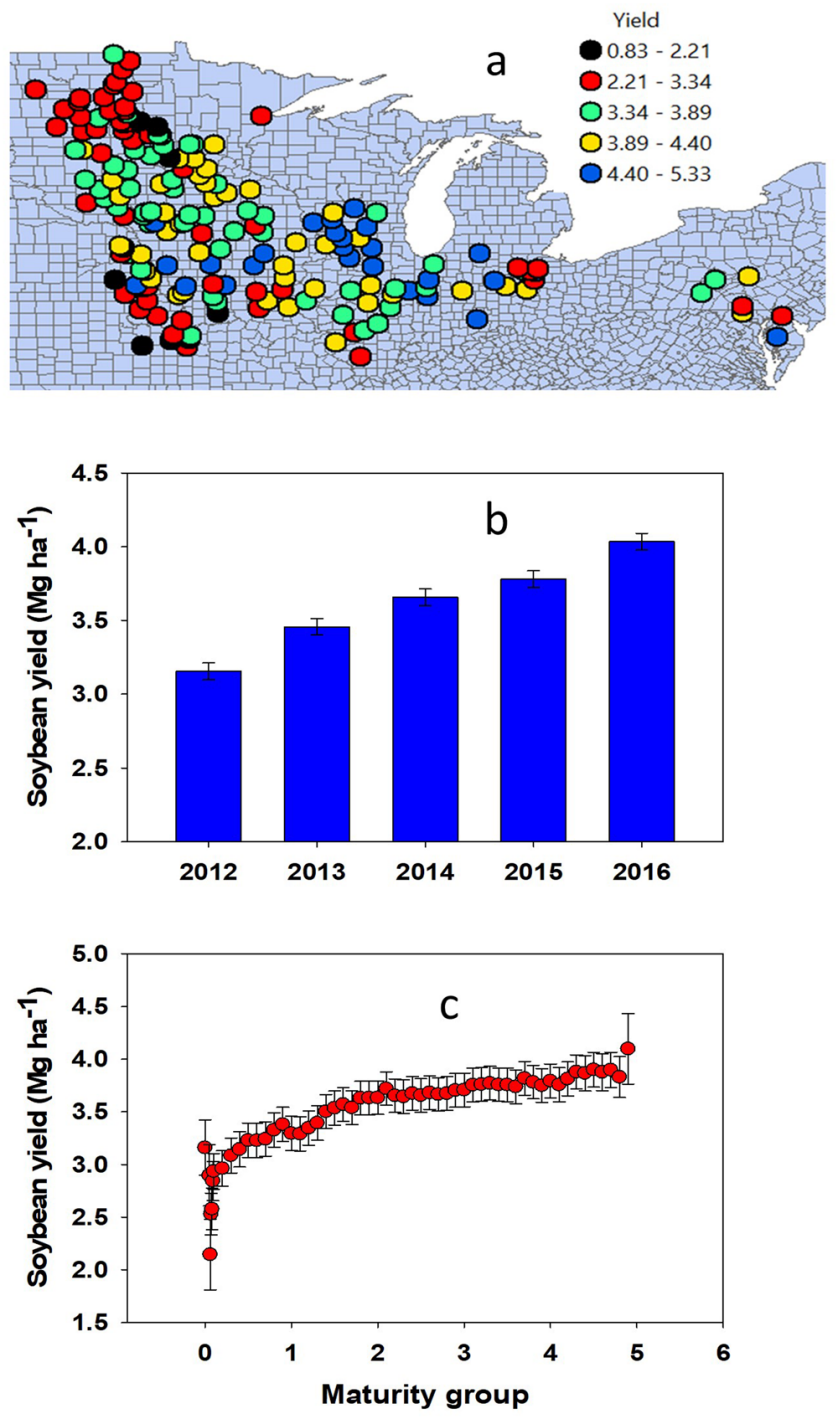
Relationship between soybean yield and location (**a**) yield at different years (**b**) and yield and maturity group (**c**) for the trial across 14 major soybean producing states in the USA for the years 2012 through 2016.

Soybean yield show a significant relationship with environment and maturity group (Fig. 8). Yield was greater in the latitude range 41–43°N compared to <41 or >43°N (Fig. 8a). No significant yield differences across east-west line (longitude) were reported, but yield tend to decrease in the extreme west (−93 to −100°W) relative to the rest of the region (Fig. 8a). Unlike AA concentration, variable by year, average yield significantly increased over the years (Fig. 8b), and with maturity group (Fig. 8c) in greater magnitude than the correlation between AA and maturity group (Fig. 6a). The similar spatial trend for yield, protein, and oil concentrations across latitude suggest that yield increase does not necessarily decrease actual protein or AA content.

### Grain Quality: Concentration versus Content (Yield)

Unlike the weak negative correlation between most of AAs and protein concentration with yield, the correlation between AAs and protein expressed per unit area (kg ha^−1^) with soybean seed yield was strong and positive (Table 4). The correlation between AA yield (unit area) with oil yield was also positive and strong.

**Table 4:** Correlation analysis among location (latitude, Lat; longitude, Long), year, amino acid, oil, protein yields (content), and seed yield for soybeans.

	Lat	Long	year	Arg.	Cyst.	Isole.	Leuc.	Lys.	Meth.	Thre.	Tryp.	Vali.	Yield	Protein
Arginine	−0.24	0.29	0.15	1.00										
	<0.0001	<0.0001	<0.0001											
Cysteine	−0.21	0.26	0.36	0.92	1.00									
	<0.0001	<0.0001	<0.0001	<0.0001										
Isoleucine	−0.21	0.27	0.35	0.97	0.94	1.00								
	<0.0001	<0.0001	<0.0001	<0.0001	<0.0001									
Leucine	−0.22	0.28	0.24	0.99	0.94	0.98	1.00							
	<0.0001	<0.0001	<0.0001	<0.0001	<0.0001	<0.0001								
Lysine	−0.21	0.28	0.21	0.99	0.92	0.97	0.99	1.00						
	<0.0001	<0.0001	<0.0001	<0.0001	<0.0001	<0.0001	<0.0001							
Methionine	−0.21	0.28	0.21	0.98	0.91	0.97	0.99	0.99	1.00					
	<0.0001	<0.0001	<0.0001	<0.0001	<0.0001	<0.0001	<0.0001	<0.0001						
Threonine	−0.22	0.30	0.18	0.99	0.92	0.96	0.99	0.99	0.98	1.00				
	<0.0001	<0.0001	<0.0001	<0.0001	<0.0001	<0.0001	<0.0001	<0.0001	<0.0001					
Tryptophan	−0.26	0.32	0.16	0.97	0.89	0.92	0.97	0.96	0.95	0.98	1.00			
	<0.0001	<0.0001	<0.0001	<0.0001	<0.0001	<0.0001	<0.0001	<0.0001	<0.0001	<0.0001				
Valine	−0.20	0.27	0.33	0.97	0.95	1.00	0.98	0.97	0.97	0.96	0.93	1.00		
	<0.0001	<0.0001	<0.0001	<0.0001	<0.0001	<0.0001	<0.0001	<0.0001	<0.0001	<0.0001	<0.0001			
Yield	−0.20	0.28	0.28	0.97	0.91	0.98	0.98	0.98	0.98	0.97	0.93	0.98	1.00	
	<0.0001	<0.0001	<0.0001	<0.0001	<0.0001	<0.0001	<0.0001	<0.0001	<0.0001	<0.0001	<0.0001	<0.0001		
Protein	−0.23	0.29	0.25	0.99	0.92	0.98	0.99	0.99	0.98	0.98	0.95	0.98	0.99	1.00
	<0.0001	<0.0001	<0.0001	<0.0001	<0.0001	<0.0001	<0.0001	<0.0001	<0.0001	<0.0001	<0.0001	<0.0001	<0.0001	
oil	−0.23	0.31	0.31	0.92	0.89	0.96	0.95	0.96	0.94	0.95	0.90	0.95	0.98	0.96
	<0.0001	<0.0001	<0.0001	<0.0001	<0.0001	<0.0001	<0.0001	<0.0001	<0.0001	<0.0001	<0.0001	<0.0001	<0.0001	<0.0001

Pearson correlation coefficients (r) and probability > |r| under null hypothesis (H0): Rho = 0. The correlation analysis had a minimum of 33,526 data points, 4 years (2012–2016) and about 120 locations each year.

**Figure 9:**
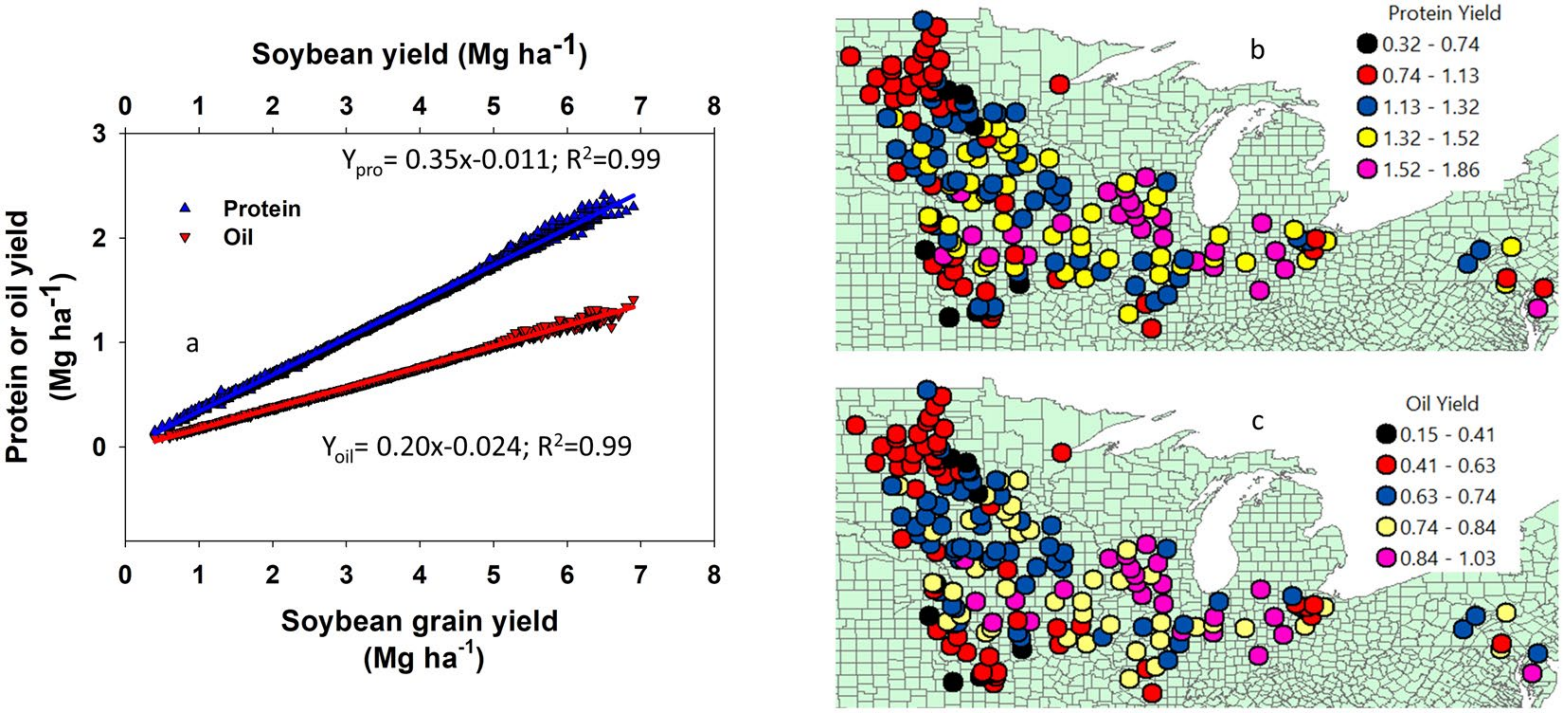
Relationship between soybean grain yield with protein and oil yields (**a**) spatial trend for protein yield (**b**) and oil yield (**c**) across 14 major soybean producing states in the USA average for the years 2012 through 2016.

A regression analysis between seed yield with oil and protein yields indicated that for a 1 Mg ha^−1^ seed yield increase, protein yield increased by 0.35 Mg ha^−1^ and oil yield improved by 0.20 Mg ha^−1^ (Fig. 9a). The obtained slope (0.35 Mg protein Mg^−1^ seed yield) reflects the average US soybean seed protein concentration. Future challenges for agronomic programs will be to identify combination of practices increasing this slope (efficiency) or seed yield or both. The spatial trend of both protein and oil yield indicate their close similarity with the yield spatial pattern (Fig. 8a–c).

The strong correlation among seed yield and quality factors expressed in a per-unit-area basis is in agreement with previous findings^22,32,38^. Rotundo *et al*.^38^ and Ray *et al*.^22^ reported an increase in protein and oil yield with increased application of fertilizer but a decline in protein concentration. Since the concentration of a particular seed quality component is dependent on other factors, it does not provide the actual amount of the seed component produced per seed, per area, or per yield. A correlation between two factors such as yield and protein concentration, therefore, might misguide reflecting that yield and quality are inversely related when that is not the case. In fact, a past review on this topic conveyed concern that the negative correlation between protein concentration and yield might hamper cultivar development^38^. Thus, future studies exploring the yield-quality relationship should focused in both as content and concentration.

## Conclusion

Analysis of multi-site-year dataset (n = 35, 101) with seed yield and quality data indicated a significant spatial autocorrelation for soybean yield and quality parameters. Variability in quality traits across regions was related to genetics, management, and environmental (G × E × M) factors. Despite a weak negative relationship between yield and AAs or protein concentration, both tended to decrease from southern to northern regions (and with the maturity group, length of the growing season). Results suggest that for each 1 Mg ha^−1^ yield increase, protein yield increased by 0.35 Mg ha^−1^ and oil yield improved by 0.20 Mg ha^−1^. Changes in G × M × E across latitudes which influence yield also affect soybean quality in a similar fashion. The positive relationship among essential AAs could provide a foundational platform for breeding and agronomic programs with the goal of focusing of improving several AAs at the same time. Future research should continue to look at the impact of different agronomic management factors on AAs (both concentration and content) and their relationship with yield and oil to better understand and identify the best management practices (BMPs) for improving both yield and quality.

## Material and Methods

Data from soybean testing programs conducted across 14 US states (Fig. 1) from 2012 to 2016 period (n = 35,101 data points) was used for our analysis. Up to twelve soybean testing regions were predefined (http://www.firstseedtests.com/map-soybean.shtml) based on location and soybean maturity group. Within a region, soybean varieties were tested in four locations, selected to represent the diversity in the region. Within a location, soybean varieties were planted on farm either in four rows of 76 cm spacing or seven rows of 34 cm spacing, by 13.7 m row length. Seed companies entered their soybean varieties within specified maturity group for the region every year. Varieties were randomized and replicated at least three times.

Soybean was planted and managed following region-specific recommendation. Planting date varied by location and year but ranged from early May to late June (Table 1). Plant stand, yield, seed moisture, oil, protein, and AA concentrations [all determined by near infrared (NIR) spectroscopy] were among variables measured. The essential and conditional essential AAs measured include Arginine, Cysteine, Isoleucine, Leucine, Lysine, Methionine, Threonine, Tryptophan, and Valine. Harvest date also varied by location and years, from late-September to mid-November. Yield and concentrations of AAs, oil, and protein were adjusted to 130 g kg^−1^ seed moisture content.

In addition to yield and seed quality composition (AAs, protein, and oil concentration), the current study included analysis on derivatives such as sum of total AA, sum of concentration of sulfur containing essential AAs (cysteine and methionine) is presented as TSAA, and the sum of the concentration of oil and protein is referred as oil + protein. The sum of total essential AAs refers to sum of the concentrations of arginine, cysteine, isoleucine, leucine, lysine, methionine, threonine, tryptophan, and valine.

As a first step, a general descriptive analysis of sum of total AA, (total AA) protein concentration, and oil + protein was conducted. Similarly, a descriptive analysis of concentration of individual AA data distribution, mean, minimum, maximum, and median values were calculated for the entire data set to highlight the variation in the data across environment and management factors.

As a second step, a spatial correlation analysis was conducted using Moran’s I in R program^39^. Spatial classification of average values of each AA was conducted in ArcMap and plots are presented for visual analysis of spatial trend across the study area. Correlation analysis of concentration of AA with latitude, longitude, and years was conducted using PROC CORR procedure of SAS^40^.

For a third step, the relationship between AAs with soybean maturity group within regions with a significant similarity in maturity group was conducted. Regions 1 to 4, 5 to 8, and 8 to 12 were identified as regions with significant similarity in maturity groups tested (Fig. 1). A comparison of the concentration of AA among the three regional groups was also conducted using PROC MIXED procedure of SAS.

As the fourth step, the interrelationship among each of the essential AAs was also conducted to understand the effect of change in one AA over the other. A similar correlation analysis was also conducted for each of the essential AAs with the rest of the seed quality traits and yield. In order to determine the actual relationship between yield and AAs; the impact of location, year, and maturity group was also analyzed.

Lastly, quality factor per ha^−1^ (AA yield ha^−1^, protein yield ha^−1^, oil yield ha^−1^) were calculated by multiplying seed yield with percentages of each quality factor. A correlation analysis was conducted at the per-unit-area basis with yield of AAs, protein, oil, and seed yield among themselves and with location and year. Regression analysis was also conducted to determine changes in protein and oil yield ha^−1^ per change in seed yield. A summary was prepared from the overall analysis of impact of location, year, and maturity group on AAs; the interrelationship and relationship between AAs and yield, and the impact of the aforementioned variables (e.g., location, year, maturity group) on yield.
